# A case of concomitant systemic lupus erythematosus and Takayasu arteritis

**DOI:** 10.1093/rap/rkaf084

**Published:** 2025-07-26

**Authors:** Lindsay N Moy, Amir Abidov, Mani Maheshwari

**Affiliations:** Division of Immunology, University of Iowa Health, Iowa City, IA, USA; Division of Immunology, University of Iowa Health, Iowa City, IA, USA; Division of Immunology, University of Iowa Health, Iowa City, IA, USA

Key message

- Autoimmune conditions are rare diagnoses; we present the case of a woman with two conditions.

Dear Editor, A 31-year-old Asian woman was diagnosed with systemic lupus erythematosus (SLE) 5 years ago after presenting with arthralgia with objective evidence of synovitis in the wrist in the setting of a positive antinuclear antibody with a titer of 1:320 in a homogenous staining pattern. Three years after diagnosis, she was found to have lupus nephritis class IV and V. She achieved remission with hydroxychloroquine (HCQ), mycophenolate mofetil (MMF), and belimumab (BEL). BEL was later stopped due to financial constraints.

Approximately 1 year off BEL, while on HCQ and MMF, she presented to the clinic with complaints of joint pain and stiffness lasting up to 12 h, occurring three to four times monthly. She also endorsed intermittent chest pressure and a sensation of skipped heartbeats. Physical examination revealed mild synovitis in the metacarpophalangeal and proximal interphalangeal joints. Labs showed rising double-stranded DNA (dsDNA) and proteinuria. Concerned about an evolving lupus flare, low-dose glucocorticoids were initiated, and plans were made to reinitiate BEL. Given her cardiac symptoms, a transthoracic echocardiogram was obtained, which revealed new moderate aortic insufficiency of unclear cause. Given concern for infective endocarditis, she was admitted for further evaluation.

Upon admission, immunosuppressive therapy was held, and she was continued on HCQ. Antiphospholipid antibodies were negative and a transesophageal echocardiogram revealed no vegetation or valvular abnormalities. Computed tomography (CT) imaging of the chest, abdomen and pelvis revealed inflammation near the aortic bifurcation, trace pericardial and pleural effusions, mild pelvic ascites, and mildly enlarged retroperitoneal lymph nodes. During hospitalization, she remained febrile (Tmax 103.3 °F) and reported recurrent episodes of chest pressure with headaches and left upper extremity paresthesias. Notably, her lupus disease activity markers remained stable despite being off immunosuppressants (Fig. 1).

**Figure 1.**
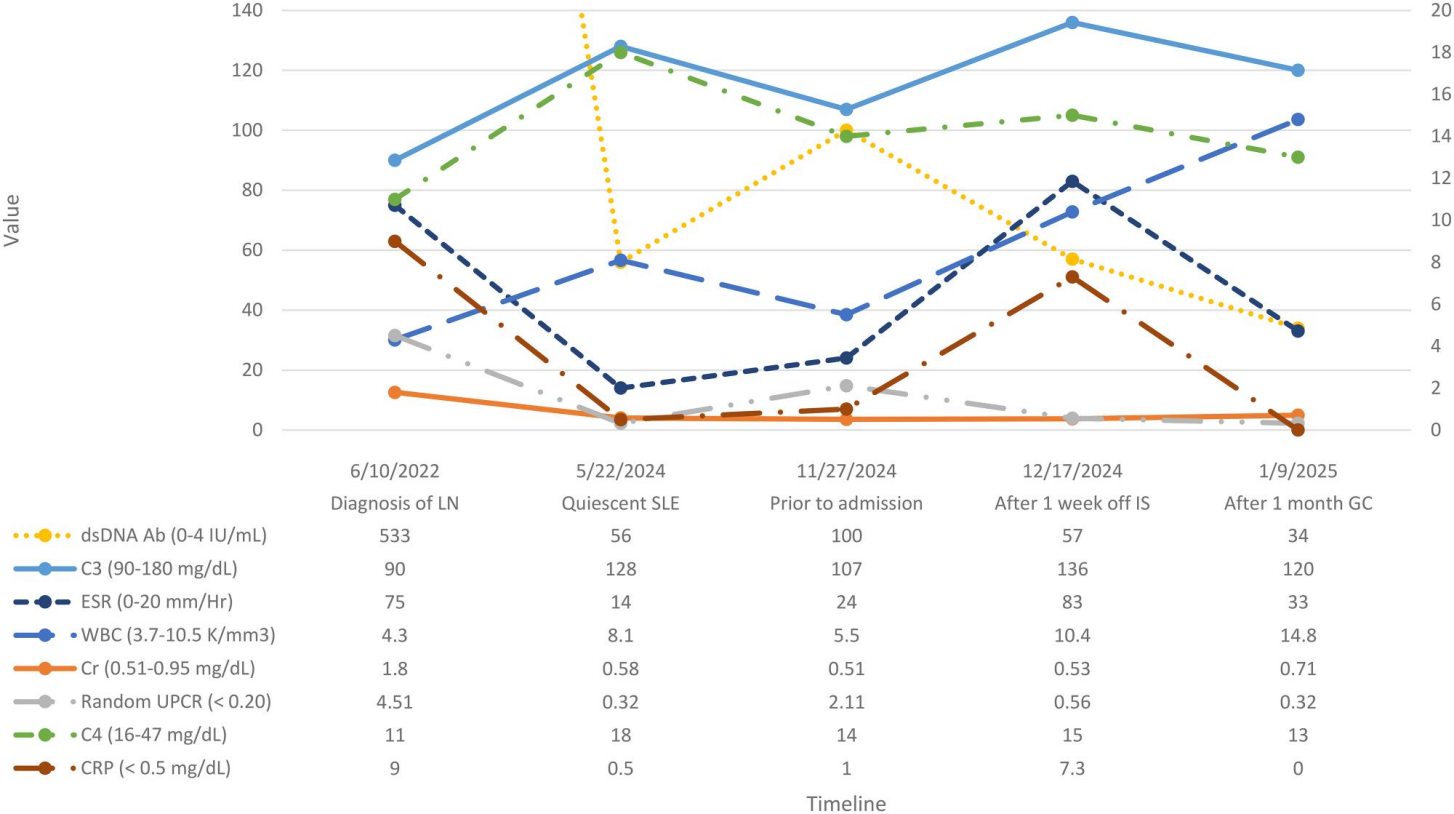
Laboratory values during various timepoints of the patient’s disease course displayed on a dual-axis chart for visual clarity. LN: lupus nephritis; SLE: systemic lupus erythematosus; IS: immunosuppressants; GC: glucocorticoids; WBC: white blood cells; Cr: creatinine; UPCR: urine protein creatinine ratio; dsDNA Ab: double stranded DNA antibody; C3: complement C3; C4: complement C4; ESR: erythrocyte sedimentation rate; CRP: C-reactive protein

The persistent systemic inflammation in the absence of active lupus nephritis, cytopenias or serositis as well as the disproportionately elevated C-reactive protein raised suspicion for an alternative diagnosis. Our differential included infection, malignancy and vasculitis. An extensive infectious workup including blood cultures, interferon gamma release assay, syphilis and evaluation for atypical infections with microbial cell-free DNA testing resulted negative. Subsequent positron emission tomography (PET)/CT scan demonstrated intense fluorodeoxyglucose (FDG) uptake in multiple large vessels, including the descending aorta, the aortic valve and the right carotid-subclavian bifurcation. FDG-avid lymphadenopathy was seen along the right common and bilateral external iliac chains (Supplementary Fig. S1). These findings were highly suggestive of a large vessel vasculitis, with the distribution and vessel wall involvement characteristic of Takayasu arteritis (TAK). There was no evidence of malignancy. Additional autoimmune testing, including antineutrophil cytoplasmic antibodies and immunoglobulin subclasses, was unremarkable.

Given the absence of active lupus features, including resolution of synovitis and stable dsDNA, complement and urinary markers while off immunosuppression, lupus aortitis was considered less likely. TAK was favored as a distinct concomitant diagnosis.

She was treated with prednisone 60 mg daily, which resolved systemic symptoms. MMF was resumed at discharge. Two months later, follow-up CT angiography showed resolution of perivascular inflammation and normalization of thoracoabdominal vasculature. She reported significant symptomatic improvement. However, discordant four-limb blood pressure readings were noted, which may be attributable to the lower sensitivity of CT angiography as compared with PET/CT. Slow glucocorticoid taper was started, and she transitioned to subcutaneous tocilizumab for steroid-sparing maintenance.

This case illustrates the rare coexistence of SLE and TAK in a single patient. Most reported cases describe TAK preceding SLE, often predating the widespread use of biologic therapies.^1^^,^^2^^,^^3^ In our patient, TAK emerged several years after a well-characterized SLE diagnosis. This temporal separation, along with distinct inflammatory features and PET/CT findings, supports a diagnosis of overlapping autoimmune disease rather than a manifestation of lupus alone.

Management of TAK in the setting of SLE presents therapeutic challenges. The 2021 ACR/Vasculitis Foundation guidelines recommend treatment of TAK with glucocorticoids, followed by conventional disease-modifying antirheumatic drugs, then tumor necrosis factor (TNF) inhibitors or interleukin-6 (IL-6) inhibitors.^4^ Our patient was already receiving MMF, which proved inadequate. TNF inhibitors were avoided due to concerns about potential SLE flare. Given emerging evidence and a favorable safety profile in lupus, IL-6 inhibition was selected.

This case highlights the potential for overlapping autoimmune syndromes and advocates for the tailored use of advanced imaging and biologic therapy in complex cases.

## Supplementary Material

rkaf084_Supplementary_Data

## Data Availability

Data are available upon request from the corresponding author.
