# Supplementary material for: A case of concomitant systemic lupus erythematosus and Takayasu arteritis
Source: Rheumatol Adv Pract. 2025 Jul 26;9(3):rkaf084. doi: 10.1093/rap/rkaf084 (PMC12311296; doi:10.1093/rap/rkaf084)
Supplement: rkaf084_Supplementary_Data [file rkaf084_supplementary_data.docx]

**Supplementary Figure S1**. PET/CT showing evidence of vasculitis with intense FDG uptake near the right carotid-subclavian bifurcation and along the bilateral external iliac chains.


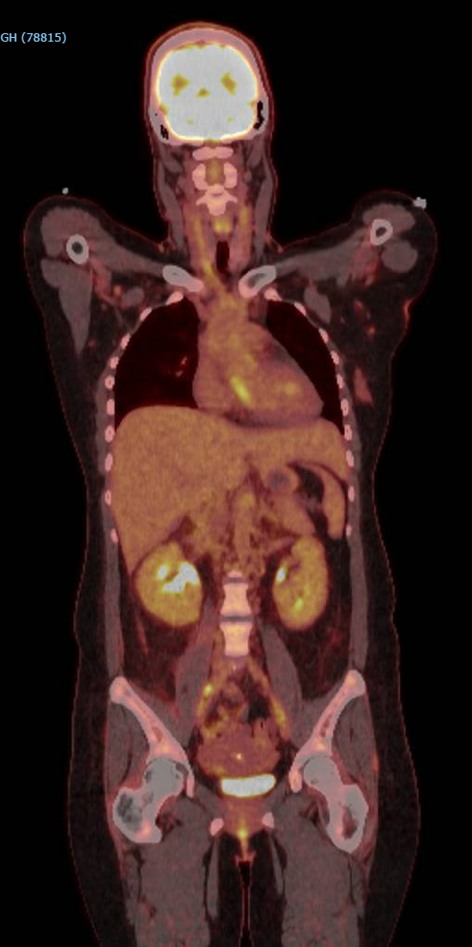


Alt text: PET/CT image showing evidence of vasculitis with intense FDG uptake near the right carotid-subclavian bifurcation and along the bilateral external iliac chains.
